# Hepatic Perivascular Epithelioid Cell Tumor (PEComa): A Case Report

**DOI:** 10.7759/cureus.75343

**Published:** 2024-12-08

**Authors:** Ismail Althunibat, Ahmad Alomari, Ahmad Habbas, Raed Atiyat, Yatinder Bains, Mehul Shah, Theresa Aquino, Yin Zhiwei

**Affiliations:** 1 Internal Medicine, Saint Michael's Medical Center, Newark, USA; 2 Internal Medicine, Henry Ford Health System, Detroit, USA; 3 Gastroenterology, Saint Michael's Medical Center, Newark, USA; 4 Interventional Radiology, Saint Michael's Medical Center, Newark, USA; 5 Pathology and Laboratory Medicine, Saint Michael's Medical Center, Newark, USA

**Keywords:** hmb 45, liver tumor, melan a, pecoma, perivascular epithelioid cell tumor, smooth muscle actin (sma)

## Abstract

Perivascular epithelioid cell tumors (PEComas) are a rare group of mesenchymal neoplasms composed of perivascular epithelioid cells. While commonly found in the kidney, uterus, and soft tissues, PEComas of the liver are exceedingly rare.

We present a case of a PEComa incidentally discovered in a 73-year-old female patient undergoing evaluation for abdominal pain. Imaging revealed an indeterminant mass in the left hepatic lobe without internal color uptake on Doppler flow. Histopathological evaluation was consistent with PEComa. The tumor was mainly composed of well-circumscribed epithelioid and spindle cell lesions with smooth muscle differentiation. Immunohistochemical staining was positive for smooth muscle actin (SMA), human melanoma black 45 (HMB 45), and Melan A.

PEComas are usually detected incidentally during workup for other reasons. Diagnosis is based on histopathological evaluation, and although most of the cases reported in the literature were evaluated after surgical resection, some of them were diagnosed after the image-guided biopsies, as we did in our case. This entity of tumors needs further studies on their natural behavior, as some malignant cases were reported. In addition, a clearer approach to diagnosis and treatment needs to be established, and more prognostication tools and radiographic characterization are needed.

## Introduction

Perivascular epithelioid cell tumors (PEComas) are very rare mesenchymal tumors that mostly affect adult ages. It usually involves the kidneys and uterus but extremely rarely involves the liver, with about 200 cases reported in the literature [1-3]. It also affects females more than males, with a male-to-female ratio of approximately 1:6 [4].

According to the World Health Organization (WHO), PEComas are mesenchymal tumors characterized by unique cells that often appear near blood vessel walls. These tumors typically exhibit markers associated with both melanocytes and smooth muscle cells. Within the PEComa family, examples include angiomyolipoma (AML), clear cell sugar tumor (CCST), lymphangioleiomyomatosis (LAM), and other tumors sharing similar histological and immunophenotypical features arising in various soft tissue and visceral locations. They described PEC as a rare cell type that shows immune reactivity to melanocytic markers, notably HMB45, and displays an epithelioid morphology with clear-acidophilic cytoplasm [5]. This article was presented at the ACG Meeting on October 28, 2024.

## Case presentation

A 73-year-old female patient presented with an asymptomatic hepatic mass while being evaluated for lower abdominal pain. Computed tomography without contrast was done initially as the patient had pre-renal acute kidney injury at the time of evaluation; it showed circumferential thickening of the sigmoid colon and calcified uterine fibroids; no lesions were detected in the liver.

**Figure 1:**
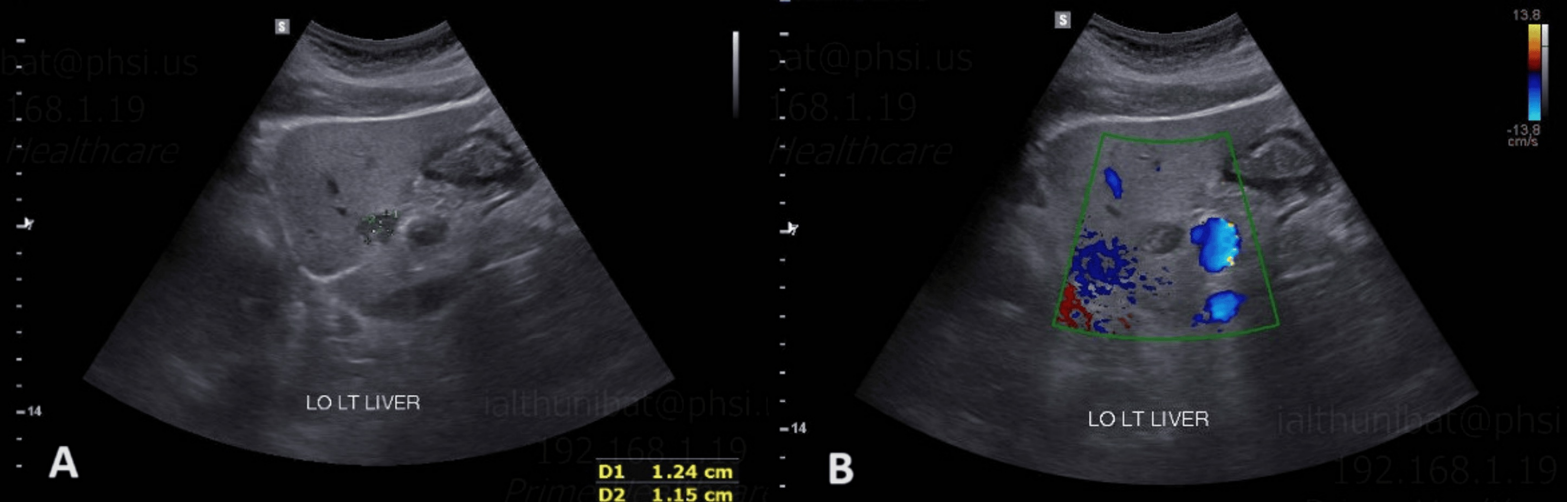
The well-circumscribed lesion in the left hepatic lobe (A), which showed hypovascularity on Doppler imaging (B).

The patient had an ultrasonographic (US) imaging of the abdomen as part of her evaluation. The imaging showed a left hepatic lobe hypovascular complex lesion measuring 1.2 x 1.2 x 1.8 cm in size (Figures 1A, 1B). The patient was discharged after her diverticulitis had been managed and planned to undergo magnetic resonance imaging (MRI) with contrast for further evaluation of the liver mass. The patient was referred to do an image-guided biopsy, and a US under anesthesia was done, which showed a 1 cm left hepatic mass adjacent to the pancreas and lesser curvature of the stomach. A CT-guided biopsy was performed following localization of the lesion site based on ultrasound and MRI findings, as the lesion was not visualized on initial CT imaging. 

**Figure 2:**
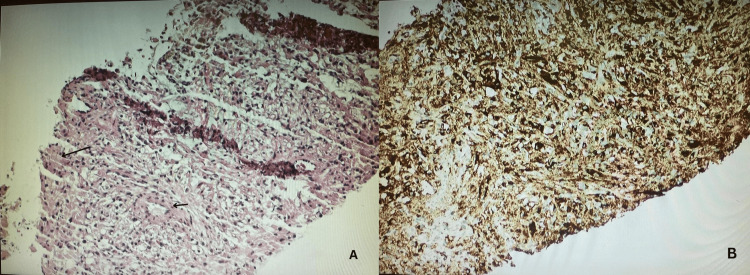
H&E staining shows hepatic tissue (long arrow), well-circumscribed epithelioid and spindle cell lesions with smooth muscle differentiation (short arrow) (A). Immunohistochemical staining shows positivity for SMA and HMB-45, indicating smooth muscle and melanocytic differentiation, respectively, consistent with PEComa (B). SMA: smooth muscle actin, HMB-45: human melanoma black 45, PEComa: perivascular epithelioid cell tumor.

Histopathological evaluation showed well-circumscribed epithelioid and spindle cells (Figure 1B). Smooth muscle differentiation was confirmed after staining with (smooth muscle actin, SMA) and Melan A. The mass also showed positivity on immunohistochemical staining with (human melanoma black 45, HMB-45), which confirms melanocytic differentiation as well, consistent with PEComa (Figure 2B). The patient was informed to be followed for further evaluation and management. 

## Discussion

PEComa comprises a spectrum of infrequent mesenchymal tumors characterized by the presence of perivascular epithelioid cells exhibiting differentiation features to melanocytes and smooth muscle cells. 

In our case, we present a challenge in diagnosis as we couldn’t perform a contrast-based CT scan and relied on the findings in the US and MRI to localize the mass. Although most of the reported cases showed that PEComas are well vascularized and show strong enhancement, the mass in our case was hypovascular on Doppler US and didn’t show a strong intensity on MRI, which made the diagnosis more difficult [6]. Diagnosis is based on histopathological evaluation, and although most of the cases reported in the literature were evaluated after surgical resection, some of them were diagnosed after image-guided biopsies, as we did in our case. 

This entity of tumors needs further studies on their natural behavior, and a clearer distinctive criterion is needed as most of them are benign, but malignant cases were reported [7]. In 2002 WHO Soft Tissue and Bone book, it was stated that PEComas demonstrating infiltrative growth along with features such as (1) tumor size > 5 cm; (2) high nuclear grade; (3) hypercellularity; (4) mitotic rate of > 1/50 high-power fields; (5) necrosis; (6) infiltration into the surrounding normal parenchyma and (7) vascular invasion should be considered malignant [8]. Nevertheless, it is important to acknowledge the existence of cases where PEComas exhibit discrepancies between histological characteristics and clinical outcomes. A case was documented where a hepatic PEComa displaying benign histological features subsequently metastasized to multiple distant sites nine years post-surgery [9]. Therefore, surgical resection with clear margins is considered the gold standard treatment modality for PEComas with surveillance due to the risk of local recurrence/metastasis [10]. Adjuvant, neoadjuvant, and immunotherapies are also used in some cases; most are nonresectable. However, based on the current literature, no definitive treatment strategy can be conclusively recommended at present [10].

## Conclusions

In conclusion, PEComas represent a rare and heterogeneous group of mesenchymal tumors characterized by the presence of perivascular epithelioid cells. While commonly found in organs, such as the kidney, uterus, and soft tissues, their occurrence in the liver is exceedingly uncommon, as demonstrated in this case of a 73-year-old female patient. The diagnosis of hepatic PEComa in this instance was challenging and required a sequential approach involving initial ultrasonographic evaluation, followed by MRI with contrast and, ultimately, CT-guided biopsy for definitive histopathological characterization.

Histologically, PEComas typically exhibit a distinctive morphology of epithelioid and spindle cells with varying degrees of smooth muscle differentiation. Immunohistochemical analysis, including positive staining for smooth muscle markers, such as SMA, Melan A, and HMB-45, further supports the diagnosis. Management strategies for PEComas remain varied and often depend on tumor size, location, and malignant potential. Given the rarity of hepatic PEComas, further studies are warranted to elucidate their natural history, optimal diagnostic approaches, and standardized treatment algorithms.
